# Modic Change Bone Marrow Neutrophils Are Activated and Degrade Cartilage Endplates

**DOI:** 10.1002/jsp2.70170

**Published:** 2026-03-18

**Authors:** Irina Heggli, Tamara Mengis, Jan Devan, Nick Herger, Mazda Farshad, Mohamed Habib, Christopher P. Ames, Oliver Distler, Aaron J. Fields, Stefan Dudli

**Affiliations:** ^1^ Center of Experimental Rheumatology, Department of Rheumatology University Hospital Zurich, University of Zurich Zurich Switzerland; ^2^ Department of Physical Medicine and Rheumatology Balgrist University Hospital, Balgrist Campus, University of Zurich Zurich Switzerland; ^3^ Department of Orthopedics Icahn School of Medicine at Mount Sinai New York New York USA; ^4^ Department of Orthopedics Balgrist University Hospital, University of Zurich Zurich Switzerland; ^5^ Department of Orthopaedic Surgery University of California San Francisco San Francisco California USA; ^6^ Department of Neurological Surgery University of California San Francisco San Francisco California USA

**Keywords:** degeneration, endplate, inflammation, modic change, neutrophils

## Abstract

**Background:**

Vertebral endplate bone marrow lesions (Modic changes, MC) are linked to vertebrogenic pain and to structural defects of the bony and cartilaginous endplates (CEP). However, the immune mechanisms that may perpetuate CEP damage in MC remain unclear.

**Objective:**

To (i) characterize neutrophils in MC bone marrow and (ii) test whether activated neutrophils can degrade human CEP tissue.

**Methods:**

In low back pain patients undergoing lumbar fusion, paired bone marrow aspirates were collected intraoperatively from an MC level and an adjacent non‐MC vertebra (intra‐patient control). MC neutrophils were characterized by bulk RNA sequencing of sorted CD45^+^CD66b^+^ cells (*n* = 7), flow cytometry (activation (CD66b); neutrophil maturation subsets), and neutrophil elastase (NE) activity in short‐term culture supernatants. To model CEP degradation, conditioned media from healthy‐donor blood neutrophils incubated ± neutrophil activator phorbol 12‐myristate 13‐acetate (PMA) (12.5 or 25 Mio cells/mL) were applied to human CEP explants (18 h). Proteoglycan loss (sGAG) and collagen loss (hydroxyproline) were quantified as release fractions, normalized to media‐only controls.

**Results:**

MC neutrophils showed an activated, pro‐inflammatory transcriptomic signature, including enrichment of calcium‐associated processes consistent with degranulation. Flow cytometry demonstrated higher CD66b intensity in MC neutrophils versus intra‐patient controls (125.4 ± 34.2%, *p* = 0.022) and a trend toward increased band neutrophils (126.7 ± 33.4%, *p* = 0.069). NE release was higher in 4/6 patients (191.2 ± 142.7%, *p* = 0.178) and correlated with band neutrophil abundance (*r* = 0.67, *p* = 0.033). PMA activation markedly increased NE activity in conditioned media and was cell‐number dependent. CEP exposure to activated neutrophil conditioned media induced significant sGAG release (25 Mio/mL: 380.1 ± 177.0%, *p* = 0.012; 12.5 Mio/mL: 123.7 ± 22.3%, *p* = 0.048), while hydroxyproline release was not significantly increased.

**Conclusions:**

Neutrophils within MC bone marrow have an activated pro‐inflammatory phenotype, and activated neutrophils can degrade CEP proteoglycans ex vivo. These findings support a potential immune‐mediated mechanism contributing to CEP weakening and indicate that MC are not just reactive changes but themselves have degenerative effects.

## Graphical abstract

Activated neutrophils accumulate in Modic‐change bone marrow and secrete neutrophil elastase, which drives cartilage endplate destruction and releases glycosaminoglycans. This neutrophil–elastase axis links inflammatory marrow lesions to structural endplate breakdown at the disc–vertebra interface.

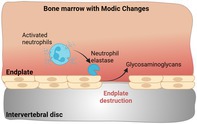

## 1 Introduction

Vertebral endplate bone marrow lesions seen on magnetic resonance imaging (MRI) as signal intensity changes (Modic changes; MC) are a source of vertebrogenic pain and associate with low back pain and disability [1, 2, 3, 4]. The endplates in MC lesions are highly innervated with PGP 9.5‐positive nerve fibers [5, 6], and ablation of the basivertebral nerve that innervates the endplates can reduce vertebrogenic pain [7]. Structural defects of both the bony and the cartilage endplate (CEP) co‐locate with MC [8, 9, 10, 11]. In longitudinal studies, endplate defects were identified as a risk factor for disc degeneration and progression of MC [9, 12], possibly by enabling a disc‐marrow cross‐talk [13, 14]. In experimental studies, endplate defects triggered MC and disc degeneration [15, 16, 17].

Despite the critical role of endplate defects in vertebrogenic pain and MC pathophysiology, the molecular and cellular mechanisms causing progressive endplate damage in MC are unclear. Whilst mechanical overloads can damage the endplates and trigger acute disc and marrow responses [18, 19], progressive endplate resorption in the MC points at perpetuating degenerative mechanisms [9]. Rat models of vertebral bone marrow lesions demonstrated that immune processes in the lesion were associated with the new occurrence of CEP damage, indicating a critical role of immune activation in CEP resorption [20]. Analysis of biopsies from human MC lesions showed vascularized granulation tissue with neutrophilic infiltrates at the bone‐disc junction and dysregulated neutrophil maturation [1, 13, 21]. In inflammatory arthritis, neutrophils are critical in joint cartilage degradation. Neutrophils in synovial fluid had an activated phenotype with increased CD66b and CD11b expression [22] and neutrophil elastase (NE) incorporated in neutrophil extracellular traps directly mediated cartilage destruction [23]. In osteoarthritic knee joints, NE concentration was increased and caused cartilage degradation through activation of the matrix metalloproteinase‐13 and through induction of apoptosis in chondrocytes [24, 25]. However, the role of neutrophils in MC and in CEP damage is unknown. Therefore, the aims of this study were (i) to phenotype MC bone marrow neutrophils, and (ii) to investigate whether activated neutrophils can degrade CEP tissue. Clarifying the role of neutrophils in MC could help understand the molecular and cellular processes causing CEP damage.

## 2 Materials and Methods

### 2.1 Study Populations and Tissue Samples

The study was conducted in accordance with the Declaration of Helsinki and approved by the local Ethics Commissions (BASEC 2017–00761, UCSF IRB#19–28 831). Informed consent was obtained from each subject.

#### 2.1.1 Study Population for the Characterization of MC Bone Marrow Neutrophils

Eighteen low back pain patients with MC undergoing lumbar spinal fusion surgery at the Balgrist University Hospital, Zurich, Switzerland, were included. Inclusion criteria were the possibility to collect two bone marrow aspirates prior to pedicle screw placement: one aspirate from a MC lesion and one from an adjacent vertebral body without MC, serving as an intra‐patient control. Exclusion criteria were prior instrumented back surgery, diagnosed infectious disease, cancer, and immunosuppressive medication. MR images were graded by a radiologist based on T1‐ and T2‐weighted sequences. Pseudo‐anonymized clinical metadata were collected: age, sex, size, weight, smoking status, fusion level, presence and type of MC type at fusion level [1], degree of disc degeneration [26], back pain and leg pain on a numeric rating scale (0–10), Oswestry Disability Index [27]. Two bone marrow aspirates (2–3 mL) were collected per patient with Jamshidi needles using the pedicle screw trajectories prior to screw insertion as described previously [28]: one aspirate from the MC lesion, and one from control bone marrow from an adjacent vertebra without MC. Aspirates were collected intraoperatively into K2‐EDTA tubes, immediately transported to the laboratory, processed within 60 min of collection, and assigned to the indicated downstream assays (Table S1).

#### 2.1.2 Study Population to Investigate Neutrophil‐Mediated CEP Degradation

CEP tissues were collected from patients undergoing anterior lumbar interbody fusion surgery for degenerative scoliosis at the University of California, San Francisco. Inclusion criteria were: (1) patient over 18 years old; (2) diagnosis of degenerative thoracolumbar spinal deformity; and (3) treatment plan including single or multilevel anterior lumbar interbody fusion of L3–S1 levels for correction of the spinal deformity. The primary exclusion criteria were pediatric patients and/or patients with a cancer diagnosis, infection, or trauma as the cause of their spinal deformity, and presence of MC at the level of CEP collection. CEP tissues collected intra‐operatively were immediately transferred to a laboratory with a sterile dissection environment wherein the CEPs were cleaned of any residual nucleus pulposus tissue, bony endplate, and blood, and stored at −20°C.

### 2.2 Chemicals and Statistical Analysis

Chemicals were purchased from Sigma‐Aldrich (Saint Luise, MO, USA) if not stated otherwise. Statistical analyses were performed using GraphPad Prism version 10.6.1, if not stated otherwise. Normal distribution was tested using the Shapiro–Wilk test. Significance level was α = 0.05.

### 2.3 MC Neutrophil Characterization

#### 2.3.1 Bulk RNA Sequencing

The bone marrow neutrophil transcriptome was analyzed in the first seven patients; neutrophils from the other eleven patients were used for subsequent functional analysis. Erythrocytes were lysed in total bone marrow aspirates with Ammonium‐Chloride‐Potassium (ACK) lysis buffer (0.15 mol/L NH_4_Cl, 0.01 mol/L KHCO_3_, 0.0001 mol/L Na_2_EDTA, pH = 7.2–7.4). Nucleated cells were stained for CD45, CD66b, and Zombie Aqua Cells for 45 min at room temperature, washed with FACS buffer (PBS without Ca^2+^ and Mg^2+^, 1% FCS) and cells positive for CD45^+^CD66b^+^ (Biolegend, London, UK) and negative for Zombie Aqua (Biolegend, London, UK) were directly sorted (FACSAria Fusion, BD, Bridgend, UK) into Qiazol. RNA was isolated using the miRNeasy Mini Kit (Qiagen, Hilden, Germany) according to the manufacturer's instructions. Libraries were prepared with SMARTer Stranded Total RNA‐Seq Kit v2—Pico Input Mammalian (Takara) and sequenced using the NovaSeq 6000 (Illumina) in single read mode (101 cycles, > 20 million reads per sample). The quality of data readings was assessed using FastQC. Adaptor sequences were removed, and four bases trimmed from each end using Trimmomatic (v0.36). Readings longer than > 30 nt were aligned to the reference genome hg38 using STAR (v2.6.0c) and counted with FeatureCounts function in the Rsubreads package. Data is available at the European Nucleotide Archive at EMBL‐EBI under accession number PRJEB61717. Differentially expressed genes (*p* < 0.01, log2fc > 0) in MC vs. intra‐patient controls were identified with edgeR (v4.6.3), using patient as a secondary factor. Overrepresentation Analysis (ORA) was performed with SUSHI [29], and clusterProfiler (v4.16.0), and terms with a false discovery rate (FDR) < 0.05 were considered. Results were visualized using a Shiny App [30].

#### 2.3.2 Flow Cytometric Phenotyping

Erythrocytes were lysed in total bone marrow aspirates with ACK lysis buffer. Cells were stained for CD45, CD66b, CD10, CD11b, and Zombie Aqua for 45 min at room temperature, washed with FACS buffer (PBS without Ca^2+^ and Mg^+^, 1% FCS) and analyzed with BD LSRFortessa Flow Cytometer. Neutrophil activation was assessed as CD66b mean fluorescence intensity of CD45^+^CD66b^+^ZombieAqua^−^ neutrophils (*n* = 10). Proportions of mature neutrophils (CD66b^+^CD10^+^CD11b^high^), immature band neutrophils (CD66b^+^CD10^−^CD11b^high^), myelocytes (CD66b^+^CD10^−^CD11b^int^), and promyelocytes (CD66b^+^CD10^−^CD11b^−^) were calculated (*n* = 7). CD66b mean fluorescence intensity (MFI) and fractions of neutrophil subsets were compared between MC and controls with paired *t*‐tests.

#### 2.3.3 NE Release

Neutrophils were isolated from bone marrow aspirates by 6% dextran sedimentation at room temperature for 45 min to remove erythrocytes, followed by purification with EasySep Human Neutrophil Isolation Kit (StemCell Technologies, Vancouver, BC, Canada) following the manufacturer's instructions (*n* = 6). For NE release assays, 25 Mio neutrophils/mL were cultured for 3 h in RPMI medium supplemented with 10% FCS in low‐attachment 96‐well plates at 37°C. Supernatants were collected by centrifugation (5 min, 500 g) and stored at −20°C until analysis. NE activity was measured using Neutrophil Elastase Activity Assay Kit (Cayman Chemical, Ann Arbour, MI, USA), and relative activity (MC vs. control) was tested against the null hypothesis (μ₀ = 100%) using a one‐sample *t*‐test.

### 2.4 CEP Resorption Model

To investigate whether neutrophils can resorb CEP, human CEP tissues were incubated with conditioned media from activated neutrophils, and collagen and proteoglycan release from CEPs was quantified in supernatants.

#### 2.4.1 Conditioned Media

Neutrophils were isolated from human whole peripheral blood of one healthy donor (20 year, female, African American, 80 kg, 160 cm, blood type 0+, no smoker) obtained from STEMCELL Technologies (Vancouver, BC, Canada) using EasySep Human Neutrophil Isolation Kit (STEMCELL Technologies, Vancouver, BC, Canada) according to manufacturer protocol. Neutrophils were incubated for 3 h at 37°C with or without 100 nM phorbol myristate acetate (PMA), a neutrophil activator, at cell densities typical for bone marrow (12.5 Mio/ml, 25 Mio/ml) [31, 32]. Supernatants were collected and frozen at −20°C until further processing. NE activity was measured in supernatants using Neutrophil Elastase Activity Assay Kit (Cayman Chemical, Ann Arbour, MI, USA) and compared between activated and non‐activated neutrophils using *t*‐tests.

#### 2.4.2 CEP Tissue Exposure to Neutrophil Supernatant

After thawing, biopsies were prepared for each CEP (6 patients; 3 biopsies per patient) using a circular punch (4‐mm diameter), and each biopsy was halved. Half‐biopsies were exposed for 18 h at 37°C on an orbital shaker to 0.75 U/mL collagenase P (positive control), to Hank's Balanced Salt Solution (HBSS; negative control), or to 180 μL conditioned media from 25 Mio/mL or 12.5 Mio/mL PMA‐activated or non‐activated neutrophils. Afterwards, exposure supernatant was collected and stored for subsequent biochemical analysis. CEP half‐biopsies were washed in PBS, blotted dry, weighed, dehydrated by lyophilization at 80°C for 2 h, re‐weighed, and dissolved with 1 mg/mL papain at 60°C overnight. Papain digests and exposure supernatant were analyzed for proteoglycan and collagen contents. Proteoglycan content was measured by quantifying sulphated glycosaminoglycans (sGAG) using a dimethylmethylene blue assay [33]. Collagen content was measured by quantifying hydroxyproline in neutralized exposure supernatant and papain digests. Exposure supernatants and papain digests were hydrolyzed overnight in 6 M HCl at 110°C, followed by neutralization with NaOH. Absolute hydroxyproline was determined using a Chloramine‐T colorimetric assay [34]. The amount of sGAG and collagen released from the half‐biopsies was calculated as the amount measured in the exposure supernatant divided by the total amount measured in the half‐biopsy and exposure supernatant (exposure supernatant + papain digest). Release of sGAG and of collagen was normalized to release in HBSS cultures (100%). To test if the release was greater than in HBSS, a one‐sample *t*‐test with (μ0 = 100%) was calculated. To test the effects of neutrophil concentrations on sGAG and collagen release, one‐way ANOVA was calculated, and group differences were compared with the Tukey Post Hoc test.

## 3 Results

### 3.1 Patient Characteristics of MC Patients at Balgrist

The average age of patients at surgery was 64.2 ± 13.6 years, 56% were female, and patients were on average overweight (body mass index = 29.0 ± 5.7) (Table S1). Average back pain was NRS 6.5 ± 2.2, leg pain NRS 5.3 ± 2.8, and ODI 46.9 ± 14.0. MC aspirates were taken from level L3 (*n* = 1), L4 (*n* = 6), L5 (*n* = 10), and S1 (*n* = 1) and were at the group level more caudal than for control aspirates (mean difference: 1.1 vertebral levels, *p* < 0.001). Predominant MC type at aspiration level was MC1 for all but one patient, where the predominant type was MC2. CEP donors (*n* = 6) were on average 66.7 ± 3.9 years old. Four were female. CEPs were obtained from L4/5 (*n* = 3) and L5/S1 (*n* = 3).

### 3.2 MC Bone Marrow Neutrophils Are Activated and Have a Pro‐Inflammatory Phenotype

**FIGURE 1:**
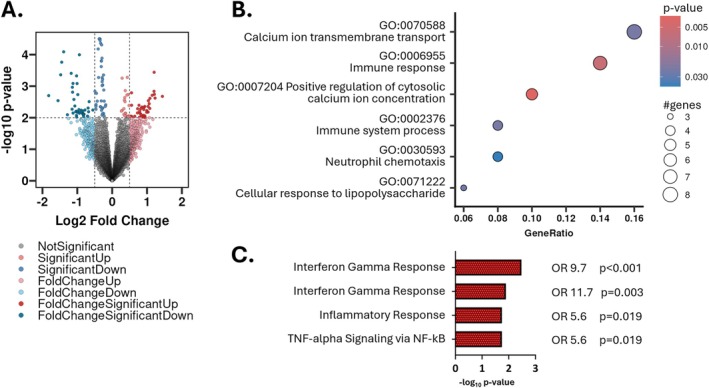
Transcriptome of neutrophils in MC bone marrow indicates activation. (A) Volcano plot of detected genes. Dashed lines indicate *p*‐value and fold‐change thresholds for the identification of differentially expressed genes. (B) Overrepresentation analysis of significantly up‐regulated genes. (C) Hallmark gene set analysis of up‐regulated genes with Enrichr. Top four gene sets are indicated.

In MC neutrophils, 57 genes were upregulated, 77 downregulated (Figure 1A, Table S2). Overrepresentation analysis revealed upregulated pathways associated with immune system activation and neutrophil chemotaxis, as well as changes in calcium ion transport, indicating increased neutrophil degranulation, a feature of activated neutrophils (Figure 1B). Hallmark gene set analysis with Enrichr of significantly upregulated genes revealed enrichment of pro‐inflammatory pathways (Figure 1C). This underscored that MC bone marrow neutrophils have an activated pro‐inflammatory transcriptome.

**FIGURE 2:**
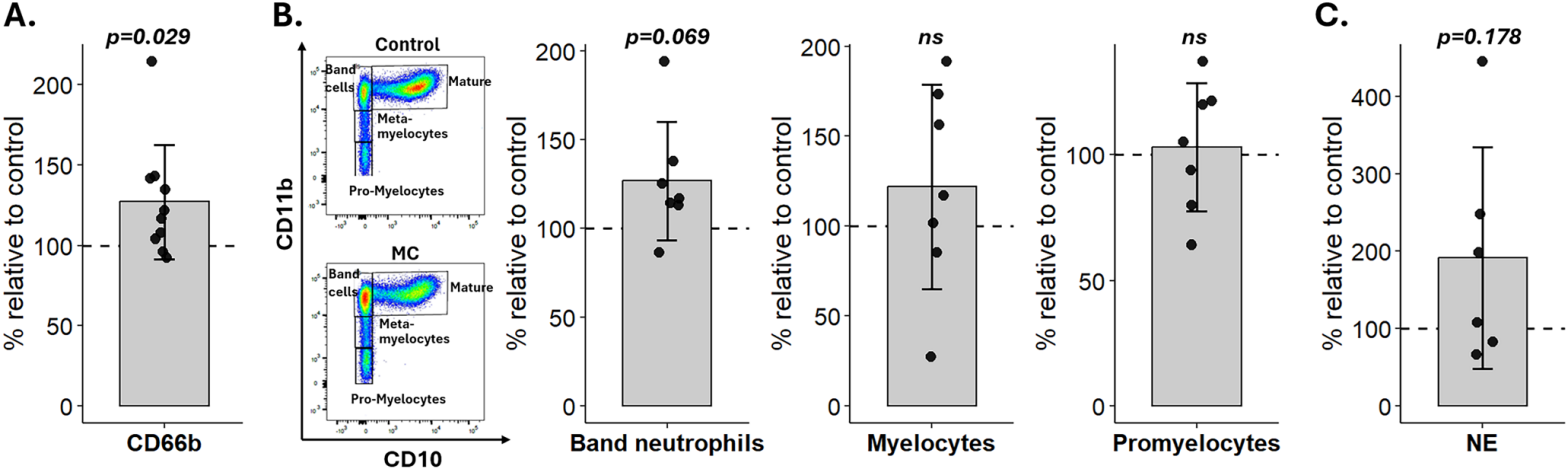
Phenotyping of neutrophils in MC bone marrow. (A) Mean CD66b expression of CD45 + CD66b + neutrophils in MC bone marrow normalized to intra‐patient control bone marrow (100%). (B) Gating strategy of representative pair of MC and control. Relative amount of CD66b + CD10‐CD11b^high^ band neutrophils, CD66b^+^CD10^−^CD11b^int^ myelocytes, and CD66b^+^CD10^−^CD11^low^ promyelocytes in MC bone marrow normalized to intra‐patient control bone marrow (100%). (C) Release of neutrophil elastase (NE) from MC neutrophils normalized to intra‐patient control bone marrow (100%).

Flow cytometric analysis revealed significantly higher intensity of the activation marker CD66b on CD45 + CD66b + neutrophils in MC bone marrow (125.4% ± 34.2%, *p* = 0.022), supporting neutrophil activation in MC (Figure 2A). Moreover, in six out of seven patients, we found increased proportions of immature band neutrophils (126.7 ± 33.4%, *p* = 0.069) (Figure 2B). Immature band neutrophils expand under inflammatory conditions [35]. This indicates an increased demand for neutrophils in the MC bone marrow and suggests a role in inflammatory MC pathomechanisms. Release of NE was higher from MC neutrophils than intra‐patient control neutrophils in four out of six patients (191.2% ± 142.7%, *p* = 0.178) (Figure 2C) and correlated with the amount of band cells (Pearson's *r* = 0.67, *p* = 0.033).

### 3.3 Activated Neutrophils Release More NE and Degrade CEP

**FIGURE 3:**
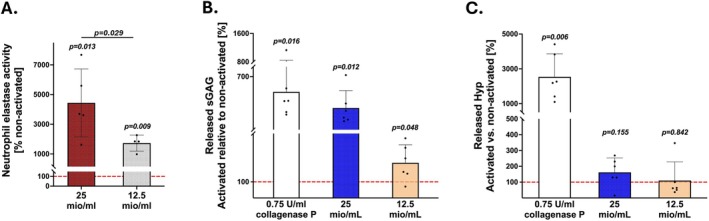
Activated neutrophils release more neutrophile elastase (NE) and degrade cartilage endplates (CEP). (A) Neutrophil elastase activity of supernatant from activated neutrophils (25 and 12.5 Mio/ml) relative to supernatants from non‐activated neutrophil (100%, red dashed line). Relative amount of sulphated glycosaminoglycans (sGAG) (B) and hydroxyproline (Hyp) (C) released from CEP tissues exposed to collagenase P (positive control, white bars) or neutrophil supernatant (blue and yellow bars). Relative sGAG and hydroxyproline release was compared to CEP cultured in control media (100%, red dashed line).

Assessment of NE activity in PMA‐activated neutrophil supernatants revealed a significant increase of NE activity relative to the corresponding non‐activated neutrophil supernatant, and the effect size was dependent on neutrophil concentration (25 Mio/ml: 4442 ± 2288%, *p* = 0.013, *n* = 5 blood donors; 12.5 Mio/ml: 1735 ± 541%, *p* = 0.009, *n* = 4 blood donors). Despite a large donor‐to‐donor variation, NE activity in PMA‐activated neutrophil supernatant was cell number dependent (25 Mio/ml vs. 12.5 Mio/ml: *p* = 0.012) (Figure 3A). To test whether activated neutrophil supernatant leads to sGAG and collagen release from CEPs, CEP tissues from six patients were exposed to activated and non‐activated neutrophil supernatant isolated from one blood donor at concentrations of 25 Mio/ml and 12.5 Mio/ml neutrophils. NE activity in the prepared supernatant was 7676% (25 Mio/ml) and 1841% (12.5 Mio/ml) relative to non‐activated neutrophils. Exposure of the CEP tissues to these neutrophil supernatants caused significant release of sGAG from the CEP tissues (25 Mio/ml: 380.1 ± 177.0%, *p* = 0.012; 12.5 Mio/ml: 123.7 ± 22.3%, *p* = 0.048), reaching nearly 70% of sGAG release by collagenase P (545.0 ± 302.8%) for the 25 Mio/ml concentration. This indicates a strong sGAG resorbent activity of activated neutrophils (Figure 3B). In contrast, there was no significant effect of neutrophil supernatant on hydroxyproline release (25 Mio/ml: 162.0 ± 90.74%, *p* = 0.155; 12.5 Mio/ml: 110.1 ± 117.9%; *p* = 0.842; positive control: 2536 ± 1321%, *p* = 0.006) (Figure 3C).

## 4 Discussion

This study shows that activated neutrophils degrade CEP tissue. Neutrophils in MC bone marrow were found to be activated, suggesting that neutrophil activation is a potential mechanism of CEP damage in MC.

At spinal levels with MC, there is increased crosstalk between the disc and the vertebral bone marrow, possibly due to endplate damage [13]. The nature of the crosstalk is unclear. Whilst MC are generally considered reactive to degenerative changes in the disc and endplate [3, 9], it is unknown if and how MC bone marrow “talks back”. Prior data indicate changes in bone marrow neutrophil composition and neutrophilic infiltrates in MC [1, 13, 21, 36], and that activated neutrophils induce articular cartilage damage in rheumatoid arthritis patients [23]. Here, we tested if MC neutrophils are activated and if activated neutrophils can degrade CEPs. To this end, we established a neutrophil‐mediated CEP damage model using PMA‐activated and non‐activated neutrophils. We exposed human CEP tissue to supernatant from PMA‐activated neutrophils at physiologically relevant densities and showed that activated neutrophils can biochemically degrade CEP tissue. This represents an accelerated CEP degradation model with orders of magnitude stronger neutrophil activation (NE release 7676% vs. 125%). For context, the relative amount of sGAG released from CEP tissues following 18 h of exposure was similar to the relative amount of sGAG lost over 20 years of natural ageing [12]. This underscores the magnitude of the biochemical degradation. sGAG‐defined mesh size is the primary factor influencing solute transport in cartilage, and sGAGs fill the large gaps (~100 nm) between collagen fibers [37]. Hence, sGAG release from the CEP is believed to increase solute transport [38]. This mechanism likely predominates during early pathogenesis, while CEPs are largely intact. Once the CEP is already damaged in MC segments, the incremental effect of sGAG loss on nutrient transport may be small. While the specific enzyme(s) responsible for neutrophil‐induced CEP tissue proteolysis need to be confirmed, our data suggest that increased NE activity may be relevant. In support of this, neutrophil‐derived NE was shown to specifically cleave aggrecan, and NE inhibition prevented neutrophil‐mediated articular cartilage damage [11]. Despite the significant effect of neutrophil supernatant on sGAG release, no significant changes in collagen release were found. This could mean that activated neutrophil supernatant does not contain enzymes with the concentration and/or specificity needed to bind to and cleave type I and type II collagen in the CEP tissue. It is also possible that collagen fragments produced during proteolysis were too large to be released from the CEP tissue. Measuring collagen fragmentation, either in bulk or spatially, is needed to assess the effect of activated neutrophil supernatant on collagen damage.

Taken together, our data suggests that activated marrow neutrophils have the potential to increase disc/marrow crosstalk in MC even without triggering overt structural damage to the CEP. The reason(s) why neutrophils in MC are activated was not a focus of this study. One potential reason is an antibacterial response to increased concentrations of intradiscal *Cutibacterium acnes* (*C. acnes*). Accumulation and activation of neutrophils have been reported in MC bone marrow, if increased numbers of *C. acnes* were found in adjacent discs [36]. In degenerated intervertebral discs from patients with low back pain or sciatica, the number of neutrophils was higher if *C. acnes* was cultured from the discs [39]. An experimental spondylodiscitis model with *Staphylococcus aureus* confirmed that infected discs recruit and activate neutrophils [40]. To confirm a similar mechanism in MC, validation in MC models with *C. acnes* is required [41].

A second potential explanation for neutrophil activation in MC is the stimulation with disc matrix fragments. During MC, the disc and endplate degenerate faster [12, 42], possibly due to increased protease activity [43]. This increases the concentration of pro‐inflammatory disc matrix fragments [43]. Whether disc fragments can activate neutrophils and whether this takes place in MC needs to be tested.

A third possible mechanism of neutrophil activation in MC is through the complement system. Neutrophils can be activated by complement factors [44], and prior studies support the complement system involvement in MC [10, 45]. The complement system is a key system for immune surveillance and can be activated by bacterial compounds as well as matrix fragments [46]. In this regard, complement system activation may mediate CEP damage in MC rather than cause MC in the first place.

Regardless of the precise mechanism(s), the data from the present study extend the concept of disc/vertebra crosstalk: MC bone marrow is not merely a reaction of bone marrow to factors within the disc, but rather MC bone marrow exhibits characteristics that may, in turn, cause degenerative CEP changes.

This study is limited by a small sample size; hence, the risk of Type I error is significant, and generalizability is restricted. While the results suggest a possible mechanism of activated neutrophils in CEP resorption, it remains to be demonstrated that activated bone marrow neutrophils can degrade CEP in vivo. Here, PMA was used to test the concept of whether activated neutrophils can degrade CEP. Since activation of neutrophils with PMA is not an MC‐relevant activation mechanism, the activation profile and mechanisms of CEP degradation may differ from activated neutrophils in MC. The CEP tissue used in this model was dead. Hence, the biological response of and interactions with endplate cells could not be investigated. Furthermore, the reason for neutrophil activation remains unknown. Existing animal models of bacterial and autoimmune MC could be utilized to answer this question [20, 41]. The functional consequences of neutrophil‐mediated sGAG release from CEPs also remain unknown. For example, it remains unknown if these CEPs have a higher permeability that facilitates an enhanced disc/marrow crosstalk and what the consequences of this enhanced crosstalk are. Lastly, although endplate damage and inflammation coincide with increased nerve fiber density in MC [5], the relevance of this novel mechanism for pain sensing remains unknown.

In summary, the results of our study showed that MC neutrophils are activated and that activated neutrophils can degrade CEP tissue. This novel mechanism demonstrates that MC are not just reactive changes visible on MRI, but that the MC bone marrow is activated and can cause degenerative CEP changes.

## Author Contributions

Study conception and design: Irina Heggli, Aaron Fields, Stefan Dudli. Tissue collection: Irina Heggli, Tamara Mengis, Jan Devan, Nick Herger, Mazda Farshad, Christopher P. Ames. Sample processing: Irina Heggli, Tamara Menigs, Jan Devan, Nick Herger, Mohamed Habib. Data analysis: Irina Heggli, Aaron J. Fields, Stefan Dudli. Manuscript drafting: Irina Heggli, Stefan Dudli. All co‐authors edited and reviewed the manuscript. Funding for this project was provided by Stefan Dudli, Aaron J. Fields, and Oliver Distler.

## Funding

This work was supported by Schweizerischer Nationalfonds zur Förderung der Wissenschaftlichen Forschung, 207989, P500PB_217823.

Gebauer Stiftung.

National Institutes of Health, R01 AR070198, P30 AR075055.

## Conflicts of Interest

The authors declare no conflicts of interest.

## Supporting information


**Table S1:** Patient demographics and assignment to neutrophil assays. Assay 1: RNA Sequencing, assay 2: flow cytometry for activation marker CD66b, assay 3: flow cytometry for maturation markers CD10, CD11, assay 4: quantification of neutrophil elastase release.

**Table S2:** Differentially expressed genes in neutrophils from Modic lesions compared to neutrophils from a control vertebra from the same patient (paired analysis). Table is ordered by increasing fdr value.

## Data Availability

The data that support the findings of this study are available from the corresponding author upon reasonable request.
