# Supplementary material for: Modic Change Bone Marrow Neutrophils Are Activated and Degrade Cartilage Endplates
Source: JOR Spine. 2026 Mar 18;9(1):e70170. doi: 10.1002/jsp2.70170 (PMC13097373; doi:10.1002/jsp2.70170)
Supplement: Supplementary file 1 — Table S1: Patient demographics and assignment to neutrophil assays. Assay 1: RNA Sequencing, assay 2: flow cytometry for activation marker CD66b, assay 3: flow cytometry for maturation markers CD10, CD11, assay 4: quantification of neutrophil elastase release. [file JSP2-9-e70170-s001.docx]

**Supplementary Table 1**. Patient demographics and assignment to neutrophil assays. Assay 1: RNA Sequencing, assay 2: flow cytometry for activation marker CD66b, assay 3: flow cytometry for maturation markers CD10, CD11, assay 4: quantification of neutrophil elastase release

| ID | Age [yr] | Sex [f/m] | Height [cm] | Weight [kg] | BMI [kg/m2] | Smoker [yes/no] | NRS LBP [0-10] | NRS leg pain [0-10] | ODI [%] | Ctrl aspiration level | MC aspiration level | Dominant MC type | Assay |
| --- | --- | --- | --- | --- | --- | --- | --- | --- | --- | --- | --- | --- | --- |
| 1 | 70 | female | 161 | 91.0 | 35.1 | yes | 9 | 9 | 66 | L4 | L5 | MC1/2 | 1 |
| 2 | 87 | female | 156 | 76.6 | 31.5 | no | 9 | 0 | 66 | L2 | L5 | MC1 | 1 |
| 3 | 80 | male | 170 | 75.0 | 26.0 | no | 9 | 8 | 48 | L3 | L4 | MC1 | 1 |
| 4 | 68 | male | 188 | 116.0 | 32.8 | no | 8 | 6 | 52 | S1 | L5 | MC1 | 1 |
| 5 | 58 | female | 153 | 90.0 | 38.4 | yes | 8 | 8 | 53 | S1 | L5 | MC1 | 1 |
| 6 | 55 | male | 182 | 106.3 | 32.1 | yes | 6 | 6 | 30 | L1 | L3 | MC1 | 1 |
| 7 | 73 | female | 162 | 79.5 | 30.3 | no | 0 | 7 | 51 | L2 | L4 | MC1 | 1 |
| 8 | 60 | female | 166 | 55.3 | 20.2 | no | 7 | 0 | 26 | L5 | L4 | MC1 | 1 |
| 9 | 47 | female | 173 | 58.0 | 19.4 | no | 5 | 2 | NA | L4 | L5 | MC1 | 2 |
| 10 | 73 | male | 178 | 77.0 | 24.3 | yes | 7 | 8 | NA | L2 | L4 | MC1 | 2 |
| 11 | 69 | male | 175 | 103.4 | 33.8 | yes | 8 | 6 | 48 | L3 | L5 | MC1 | 2 |
| 12 | 58 | female | 156 | 63.7 | 26.2 | yes | 5 | 6 | 16 | L4 | L5 | MC2 | 2, 3 |
| 13 | 35 | female | NA | NA | NA | NA | NA | NA | NA | L2 | L4 | MC1 | 2, 3, 4 |
| 14 | 52 | female | 162 | 82.0 | 31.2 | yes | 6 | 5 | 54 | L5 | S1 | MC1 | 2, 3, 4 |
| 15 | 81 | female | 153 | 53.8 | 23.0 | no | 7 | 8 | 45 | L4 | L5 | MC1 | 2, 3, 4 |
| 16 | 44 | male | 178 | 78.0 | 24.6 | yes | 4 | 2 | 60 | L4 | L5 | MC1 | 2, 3, 4 |
| 17 | 69 | male | 172 | 76.0 | 25.7 | no | 6 | 4 | 42 | L2 | L4 | MC1 | 2, 3, 4 |
| 18 | 77 | male | 183 | 130 | 38.8 | no | NA | NA | NA | L4 | L5 | MC1/3 | 2, 3, 4 |
| **mean** | **64.2** | **56%** | **168.7** | **83.0** | **29.0** | **47%** | **6.5** | **5.3** | **46.9** |  |  |  |  |
| sd | 13.6 | female | 10.8 | 20.6 | 5.7 | smoker | 2.2 | 2.8 | 14.0 |  |  |  |  |
