# Supplementary material for: Modic Change Bone Marrow Neutrophils Are Activated and Degrade Cartilage Endplates
Source: JOR Spine. 2026 Mar 18;9(1):e70170. doi: 10.1002/jsp2.70170 (PMC13097373; doi:10.1002/jsp2.70170)
Supplement: Supplementary file 2 — Table S2: Differentially expressed genes in neutrophils from Modic lesions compared to neutrophils from a control vertebra from the same patient (paired analysis). Table is ordered by increasing fdr value. [file JSP2-9-e70170-s002.docx]

Supplementary Table 2. Differentially expressed genes in neutrophils from Modic lesions compared to neutrophils from a control vertebra from the same patient (paired analysis). Table is ordered by increasing fdr value.

| gene_name | description | log2Ratio | pValue | fdr |
| --- | --- | --- | --- | --- |
| MT-ND5 | mitochondrially encoded NADH:ubiquinone oxidoreductase core subunit 5 | -0.34853942 | 3.049E-05 | 0.13500137 |
| MT-ND2 | mitochondrially encoded NADH:ubiquinone oxidoreductase core subunit 2 | -0.37370406 | 3.0946E-05 | 0.13500137 |
| MT-ATP6 | mitochondrially encoded ATP synthase membrane subunit 6 | -0.34730598 | 3.557E-05 | 0.13500137 |
| MT-ND4 | mitochondrially encoded NADH:ubiquinone oxidoreductase core subunit 4 | -0.33041117 | 4.3628E-05 | 0.13500137 |
| AC092139.3 | novel transcript | -0.30228687 | 4.9084E-05 | 0.13500137 |
| MT-ND4L | mitochondrially encoded NADH:ubiquinone oxidoreductase core subunit 4L | -0.40161796 | 6.6327E-05 | 0.15202174 |
| PRDX2 | peroxiredoxin 2 | -1.39101552 | 8.1484E-05 | 0.16008061 |
| CBWD6 | COBW domain containing 6 | -0.93951896 | 0.00010101 | 0.17363479 |
| MT-CYB | mitochondrially encoded cytochrome b | -0.29150291 | 0.00012963 | 0.19807313 |
| MT-CO2 | mitochondrially encoded cytochrome c oxidase II | -0.309323 | 0.00015932 | 0.19911858 |
| MT-ND1 | mitochondrially encoded NADH:ubiquinone oxidoreductase core subunit 1 | -0.31027266 | 0.00016093 | 0.19911858 |
| RSC1A1 | regulator of solute carriers 1 | -1.44694317 | 0.00017375 | 0.19911858 |
| MTRNR2L6 | MT-RNR2 like 6 | -0.31284973 | 0.00023981 | 0.25368194 |
| AC090517.4 | zinc finger protein 280D | 1.20404409 | 0.00036333 | 0.34909721 |
| RGPD1 | RANBP2 like and GRIP domain containing 1 | -1.17458565 | 0.00039266 | 0.34909721 |
| GMPR | guanosine monophosphate reductase | -1.47663747 | 0.00043652 | 0.34909721 |
| MT-CO1 | mitochondrially encoded cytochrome c oxidase I | -0.2477144 | 0.00044246 | 0.34909721 |
| NAXE | NAD(P)HX epimerase | -1.02135402 | 0.00047248 | 0.34909721 |
| ZFP36L1 | ZFP36 ring finger protein like 1 | 0.43384767 | 0.00052646 | 0.34909721 |
| ZFHX3 | zinc finger homeobox 3 | -0.39012512 | 0.00053236 | 0.34909721 |
| MT-CO3 | mitochondrially encoded cytochrome c oxidase III | -0.28834398 | 0.00053498 | 0.34909721 |
| FCGR3B | Fc fragment of IgG receptor IIIb | 0.28552791 | 0.00055847 | 0.34909721 |
| MTRNR2L12 | MT-RNR2 like 12 | -0.27369674 | 0.00060944 | 0.36439511 |
| MT-ND3 | mitochondrially encoded NADH:ubiquinone oxidoreductase core subunit 3 | -0.2872681 | 0.00072464 | 0.41522037 |
| GALNT9 | polypeptide N-acetylgalactosaminyltransferase 9 | -1.12333013 | 0.00082219 | 0.43715615 |
| HSPA1B | heat shock protein family A (Hsp70) member 1B | -1.00604258 | 0.0008265 | 0.43715615 |
| MTRNR2L8 | MT-RNR2 like 8 | -0.27096225 | 0.00092076 | 0.46897539 |
| MTRNR2L1 | MT-RNR2 like 1 | -0.28878858 | 0.00095924 | 0.47112604 |
| TNKS1BP1 | tankyrase 1 binding protein 1 | -1.14197488 | 0.00108438 | 0.51421971 |
| ARG1 | arginase 1 | -0.23993879 | 0.00132597 | 0.60738447 |
| IFI30 | IFI30 lysosomal thiol reductase | 0.37654241 | 0.00138475 | 0.60738447 |
| ITPKA | inositol-trisphosphate 3-kinase A | 1.20033878 | 0.00145613 | 0.60738447 |
| AC022400.5 | novel transcript | -0.49366865 | 0.00145751 | 0.60738447 |
| SIGLEC6 | sialic acid binding Ig like lectin 6 | -1.13581775 | 0.00155122 | 0.62742221 |
| GDPD3 | glycerophosphodiester phosphodiesterase domain containing 3 | 0.55533603 | 0.00162287 | 0.63764767 |
| STUM | stum, mechanosensory transduction mediator homolog | -0.45109476 | 0.00168777 | 0.64472625 |
| TNNT3 | troponin T3, fast skeletal type | 1.21109318 | 0.00181979 | 0.65299848 |
| IFIT3 | interferon induced protein with tetratricopeptide repeats 3 | 0.4606783 | 0.00184669 | 0.65299848 |
| SHE | Src homology 2 domain containing E | -0.98485937 | 0.00185389 | 0.65299848 |
| GBP1 | guanylate binding protein 1 | 0.42276756 | 0.00190819 | 0.65299848 |
| DEFA1B | defensin alpha 1B | 1.14806881 | 0.00195015 | 0.65299848 |
| AC087721.2 | novel protein | -1.82103093 | 0.00199432 | 0.65299848 |
| ARPIN-AP3S2 | ARPIN-AP3S2 readthrough | 1.44086753 | 0.0021102 | 0.67486984 |
| SLC8A1 | solute carrier family 8 member A1 | 0.36098926 | 0.00230395 | 0.72008761 |
| AL139300.1 | novel protein | 1.23094047 | 0.00239128 | 0.72862476 |
| TMEM184A | transmembrane protein 184A | 1.08434695 | 0.00243723 | 0.72862476 |
| AL132671.2 | novel protein, GOPC-ROS1 readthrough | -1.09724666 | 0.00263895 | 0.77214413 |
| FFAR2 | free fatty acid receptor 2 | 0.35243404 | 0.00279248 | 0.77619537 |
| AL132780.3 | novel protein | -1.55326655 | 0.00288064 | 0.77619537 |
| IGHG2 | immunoglobulin heavy constant gamma 2 (G2m marker) | 1.0389722 | 0.00288932 | 0.77619537 |
| MT-ATP8 | mitochondrially encoded ATP synthase membrane subunit 8 | -0.31210158 | 0.00294443 | 0.77619537 |
| TJAP1 | tight junction associated protein 1 | -0.42671414 | 0.00299068 | 0.77619537 |
| TMEM170B | transmembrane protein 170B | -0.22890421 | 0.00299466 | 0.77619537 |
| PPP1R3F | protein phosphatase 1 regulatory subunit 3F | -0.65945066 | 0.00309505 | 0.77619537 |
| KCNK6 | potassium two pore domain channel subfamily K member 6 | 0.36925457 | 0.00310433 | 0.77619537 |
| CHRNA10 | cholinergic receptor nicotinic alpha 10 subunit | 1.06274866 | 0.00360465 | 0.88519949 |
| PTMS | parathymosin | -0.46332302 | 0.0038268 | 0.91682158 |
| ANXA1 | annexin A1 | -0.18446489 | 0.00390574 | 0.91682158 |
| IFIT2 | interferon induced protein with tetratricopeptide repeats 2 | 0.55773009 | 0.00403651 | 0.91682158 |
| CISH | cytokine inducible SH2 containing protein | 0.76934235 | 0.00403799 | 0.91682158 |
| AC099489.1 | novel lipoprotein amino terminal region containing protein | 0.48302467 | 0.00411043 | 0.91682158 |
| CA1 | carbonic anhydrase 1 | -1.07054776 | 0.00425633 | 0.91682158 |
| AL356273.3 | TEC | -0.46591711 | 0.00437005 | 0.91682158 |
| PAG1 | phosphoprotein membrane anchor with glycosphingolipid microdomains 1 | -0.19515626 | 0.00438462 | 0.91682158 |
| CLDN11 | claudin 11 | 1.02963423 | 0.00440734 | 0.91682158 |
| AC104109.3 | novel protein | 0.93439841 | 0.00441807 | 0.91682158 |
| RPS10-NUDT3 | RPS10-NUDT3 readthrough | -0.33796419 | 0.00448071 | 0.91682158 |
| IRS2 | insulin receptor substrate 2 | -0.32706247 | 0.00456193 | 0.91682158 |
| OLFML2A | olfactomedin like 2A | 0.85219511 | 0.00460011 | 0.91682158 |
| TMC1 | transmembrane channel like 1 | 0.90727982 | 0.00474233 | 0.92698824 |
| SLC36A4 | solute carrier family 36 member 4 | -0.26707257 | 0.00491985 | 0.92698824 |
| CORO7-PAM16 | CORO7-PAM16 readthrough | -0.69056531 | 0.00494415 | 0.92698824 |
| ZNF107 | zinc finger protein 107 | -0.29051415 | 0.00505529 | 0.92698824 |
| PRG2 | proteoglycan 2, pro eosinophil major basic protein | 1.07977941 | 0.00508757 | 0.92698824 |
| IL5RA | interleukin 5 receptor subunit alpha | 0.82229563 | 0.00518147 | 0.92698824 |
| CARD16 | caspase recruitment domain family member 16 | 0.33592621 | 0.00518542 | 0.92698824 |
| BBS1 | Bardet-Biedl syndrome 1 | 0.7311759 | 0.00519038 | 0.92698824 |
| ITGB3 | integrin subunit beta 3 | -1.14940722 | 0.00538134 | 0.93543478 |
| DHCR7 | 7-dehydrocholesterol reductase | -0.46428619 | 0.00540023 | 0.93543478 |
| TMEM39B | transmembrane protein 39B | 0.90775721 | 0.00551351 | 0.93543478 |
| LAMB2 | laminin subunit beta 2 | -0.9534863 | 0.00552561 | 0.93543478 |
| ANK1 | ankyrin 1 | -0.78040495 | 0.00571242 | 0.93543478 |
| TBL3 | transducin beta like 3 | -0.55494015 | 0.00572581 | 0.93543478 |
| NEO1 | neogenin 1 | -0.88424845 | 0.00580946 | 0.93543478 |
| TFRC | transferrin receptor | -0.30431259 | 0.0058363 | 0.93543478 |
| ITGA5 | integrin subunit alpha 5 | 0.28526529 | 0.00589356 | 0.93543478 |
| PAX3 | paired box 3 | 0.99402262 | 0.00596269 | 0.93543478 |
| CA2 | carbonic anhydrase 2 | -0.83762564 | 0.00614893 | 0.93543478 |
| TNFAIP2 | TNF alpha induced protein 2 | 0.26418495 | 0.00617567 | 0.93543478 |
| MATN2 | matrilin 2 | -1.0277053 | 0.00622584 | 0.93543478 |
| ALOX15 | arachidonate 15-lipoxygenase | 0.78986142 | 0.00622636 | 0.93543478 |
| HSPH1 | heat shock protein family H (Hsp110) member 1 | -0.67697855 | 0.00647204 | 0.93543478 |
| PF4 | platelet factor 4 | -1.042201 | 0.00647766 | 0.93543478 |
| TMC2 | transmembrane channel like 2 | 1.03011475 | 0.00663493 | 0.93543478 |
| SPTB | spectrin beta, erythrocytic | -0.89222443 | 0.00668826 | 0.93543478 |
| CAVIN2 | caveolae associated protein 2 | -0.96509277 | 0.00670149 | 0.93543478 |
| HEMGN | hemogen | -0.9927277 | 0.00671694 | 0.93543478 |
| ANKRD39 | ankyrin repeat domain 39 | 0.98964312 | 0.00672095 | 0.93543478 |
| AC008763.2 | novel transcript | 0.72635929 | 0.00706114 | 0.93543478 |
| AL137783.1 | TEC | -1.12139792 | 0.00713037 | 0.93543478 |
| EMC3 | ER membrane protein complex subunit 3 | 0.38346005 | 0.00717997 | 0.93543478 |
| AC104581.2 | novel transcript | -0.93790626 | 0.00722393 | 0.93543478 |
| UNC45A | unc-45 myosin chaperone A | -0.24595453 | 0.00735696 | 0.93543478 |
| EIF3B | eukaryotic translation initiation factor 3 subunit B | -0.27396032 | 0.00736269 | 0.93543478 |
| S100B | S100 calcium binding protein B | 0.805155 | 0.00736807 | 0.93543478 |
| FAM104A | family with sequence similarity 104 member A | 0.3771992 | 0.00742573 | 0.93543478 |
| FAM9C | family with sequence similarity 9 member C | -0.84913376 | 0.00747922 | 0.93543478 |
| NUTM2G | NUT family member 2G | -0.82625854 | 0.0076144 | 0.93543478 |
| MYH10 | myosin heavy chain 10 | -0.80785533 | 0.00774165 | 0.93543478 |
| CXCR2 | C-X-C motif chemokine receptor 2 | 0.33714471 | 0.00785213 | 0.93543478 |
| CCL3L1 | C-C motif chemokine ligand 3 like 1 | 0.8292847 | 0.0079832 | 0.93543478 |
| DGCR6 | DiGeorge syndrome critical region gene 6 | -0.82935508 | 0.0080447 | 0.93543478 |
| AC008403.1 | novel protein | -1.28288139 | 0.00806148 | 0.93543478 |
| ADGRF1 | adhesion G protein-coupled receptor F1 | -0.29034836 | 0.00831933 | 0.93543478 |
| SMIM35 | small integral membrane protein 35 | 0.90079084 | 0.00839738 | 0.93543478 |
| EPB42 | erythrocyte membrane protein band 4.2 | -1.0226651 | 0.00844273 | 0.93543478 |
| CDK5R1 | cyclin dependent kinase 5 regulatory subunit 1 | 0.39061113 | 0.00862929 | 0.93543478 |
| FAM126A | family with sequence similarity 126 member A | 0.36325533 | 0.00870843 | 0.93543478 |
| IL1B | interleukin 1 beta | 0.47517331 | 0.00870995 | 0.93543478 |
| MEF2B | myocyte enhancer factor 2B | 0.9381936 | 0.00876599 | 0.93543478 |
| INHBA | inhibin subunit beta A | -0.23091842 | 0.00881005 | 0.93543478 |
| SLC4A1 | solute carrier family 4 member 1 (Diego blood group) | -0.70602948 | 0.00893086 | 0.93543478 |
| SPIN3 | spindlin family member 3 | 0.535443 | 0.00900004 | 0.93543478 |
| SLC7A7 | solute carrier family 7 member 7 | 0.64943488 | 0.00902956 | 0.93543478 |
| AC104389.5 | novel transcript | 0.56201584 | 0.00912921 | 0.93543478 |
| ORAI3 | ORAI calcium release-activated calcium modulator 3 | 0.49524933 | 0.00923904 | 0.93543478 |
| TFR2 | transferrin receptor 2 | -0.81906545 | 0.00925634 | 0.93543478 |
| CYSLTR1 | cysteinyl leukotriene receptor 1 | 0.43939264 | 0.00931927 | 0.93543478 |
| IL21R | interleukin 21 receptor | 0.85044519 | 0.00946131 | 0.93543478 |
| CEP68 | centrosomal protein 68 | 0.45121717 | 0.00955813 | 0.93543478 |
| HBD | hemoglobin subunit delta | -1.01874127 | 0.00957958 | 0.93543478 |
| CD274 | CD274 molecule | 0.89481313 | 0.00959122 | 0.93543478 |
| AC245100.7 | TEC | 0.7835479 | 0.00966319 | 0.93543478 |
| AC009163.5 | novel TMEM170A-CFDP1 readthrough protein | 0.94718543 | 0.00992023 | 0.93543478 |
